# Association Between BMI Z-Score and Postoperative Complications in Pediatric Neuromuscular Scoliosis Surgery

**DOI:** 10.1177/21925682261430676

**Published:** 2026-03-08

**Authors:** Alexander K. Mihas, Dion Birhiray, Chun Wai Hung, Lorenzo R. Deveza, Dallas Vanorny, Frank Gerow, Darrell Hanson, Benny Dahl

**Affiliations:** 1 Joseph Barnhart Department of Orthopedic Surgery, Baylor College of Medicine, Houston, TX, USA; 2 Georgetown University School of Medicine, Washington, DC, USA; 3 Department of Orthopedic Surgery, Texas Children’s Hospital, , Houston, TX, USA; 4 Spine Unit, Department of Orthopaedic Surgery, Rigshospitalet and University of Copenhagen, Copenhagen, Denmark

**Keywords:** neuromuscular scoliosis, BMI z-score, pediatric spinal instrumentation

## Abstract

**Study Design:**

Single-center retrospective review.

**Objective:**

To investigate the association between BMI Z-scores and postoperative complications in pediatric patients with neuromuscular scoliosis undergoing spinal instrumentation.

**Methods:**

Pediatric patients who underwent spinal instrumentation for neuromuscular scoliosis from July 2012 to June 2016 with a minimum 2-year follow-up were included. BMI and BMI Z-scores were calculated, and patients were stratified into CDC-defined BMI-for-age percentiles: underweight (<5th percentile), normal (5th to <85th percentile), overweight (85th to <95th percentile), and obese (≥95th percentile). Logistic regression assessed associations of BMI, BMI Z-score, and BMI groups with postoperative complications.

**Results:**

In total, 147 patients were included. Average follow-up was 3.2 ± 1.4 years. The most common diagnosis was cerebral palsy (39.5%). Complications occurred in 65 (44.2%) patients: 32 (21.8%) had a surgical site infection (SSI), 10 (6.8%) developed pneumonia, and 24 (16.3%) required return to the operating room within 90 days. Higher BMI Z-score was associated with increased odds of deep SSI (OR = 1.50, *P* = 0.039), 30-day readmission (OR = 1.45, *P* = 0.045), and reoperation within 90 days (OR = 1.52, *P* = 0.026), and decreased odds of pneumonia (OR = 0.77, *P* = 0.041). Similar associations were seen for BMI.

**Conclusion:**

Higher BMI Z-scores were associated with increased odds of deep SSI, readmission within 30 days, and return to the operating room within 90 days. Lower BMI Z-scores were associated with increased risk of developing pneumonia; however, this should be interpreted with caution as these patients had baseline pulmonary comorbidities. BMI Z-scores may be a useful adjunct for preoperative risk stratification in pediatric neuromuscular scoliosis patients undergoing spinal instrumentation.

**Level of Evidence:**

Prognostic Level III.

## Introduction

Surgical correction of scoliosis in pediatric patients with severe curves is often necessary for slowing curve progression, preventing cardiopulmonary compromise, improving cosmesis, and reducing pain. While surgical intervention is highly effective in addressing the consequences of severe scoliosis, it is important to consider the different subtypes of spinal deformity, which include idiopathic, congenital, neuromuscular, and syndromic scoliosis. Among these, neuromuscular scoliosis is associated with the highest rates of complications, often attributable to more severe comorbidities in these patients.^1,2^ Complication rates following spinal instrumentation in pediatric scoliosis are reported to be as high as 10.1%, but for neuromuscular scoliosis, the reported rates are between 24% to 74%.^1,2,3,4^

Since there are higher complication rates associated with surgical management of neuromuscular scoliosis, efforts are focused on identifying associated risk factors. The main complications are dominated by pulmonary issues, implant failure and pseudoarthrosis, and infections.^5^ Due to the high risk for wound complications and surgical site infections (SSIs) after pediatric scoliosis surgery, body mass index (BMI) has been identified as a possible modifiable patient factor for reducing this risk preoperatively.^6,7^ Although several studies have examined this issue in pediatric scoliosis,^8,9,10,11^ there is a paucity of literature on the association between BMI and postoperative complications in neuromuscular scoliosis. Furthermore, most of these prior studies in neuromuscular scoliosis have used BMI or categories based on BMI percentiles.^7,8,10^ BMI Z-scores are standardized scores adjusted for child age and sex, and use of a standardized score as a continuous variable may be more relevant in assessing associations with postoperative complications.^12^ To our knowledge, no study has examined the relationship between age- and sex-adjusted BMI Z-scores with postoperative complications in neuromuscular scoliosis. Therefore, the objective of the study is to investigate the association between BMI Z-scores and the risk of postoperative complications in this patient population after undergoing spinal instrumentation.

## Methods

Institutional review board approval was obtained for this retrospective cohort study. We identified and reviewed all patients with neuromuscular scoliosis who underwent spinal instrumentation at a single institution from July 2012 to June 2016. Inclusion criteria consisted of patients with a diagnosis of neuromuscular scoliosis, defined as scoliosis associated with any neuromuscular disorder (eg, cerebral palsy, muscular dystrophy, etc.). All patients with less than 2 years of follow-up and those aged 18 years or older were excluded. Procedures were performed at a tertiary pediatric referral center by fellowship-trained pediatric orthopaedic and spine surgeons. Perioperative management, including anesthesia, antibiotic prophylaxis, and postoperative care pathways, followed institutional standards in place during the study period. Surgeon-specific volume effects were not evaluated, given the retrospective design and limited power for surgeon-level comparisons.

### Variables

Patient characteristics included age, sex, body mass index (BMI), and diagnosis. BMI Z-scores and weight Z-scores were computed using the Centers for Disease Control (CDC) growth charts for children from birth to 20 years in the United States.^13^ BMI categories were based on CDC definitions: underweight (<5th percentile), normal (5th to <85th percentile), overweight (85th to <95th percentile), and obese (≥95th percentile).^12,13^ CDC BMI categories were defined using BMI-for-age percentiles (rather than absolute BMI thresholds), consistent with standard pediatric growth assessment. Outcomes assessed in this study included 30-day mortality, 1-year mortality, readmission within 30 days, return to operating room within 90 days, return to the emergency room within 90 days, superficial and deep surgical site infection (SSI) as defined by the CDC,^14^ pneumonia within 30 days, transient loss of intraoperative neuromonitoring signals, and failure of instrumentation within 1 year.

### Statistical Analysis

Categorical variables were described as percentages, and continuous variables were described as means with standard deviation. Normality of continuous variables was assessed via the Shapiro-Wilk test. Chi-square and Fisher’s Exact tests were used to compare categorical variables, and independent sample t-tests were used to compare continuous variables. Logistic regression was performed to determine the association of BMI and BMI Z-score with various complications via odds ratios with 95% confidence intervals (CI). Logistic regression was also performed to determine the association between BMI group and complications. Logistic regression probability curves were created to predict the probability of deep SSI and pneumonia as a function of BMI and BMI z-scores. All statistics were computed using RStudio version 2023.12.1 + 402 (RStudio, Boston, MA). *P*-values of <0.05 were considered statistically significant.

## Results

### Demographics & Complications

A total of 147 patients met inclusion criteria and were included in the study. Average follow-up was 3.2 ± 1.4 years (1184 ± 501 days). Average age was 13.2 ± 3.5 years and 83 (56.5%) of patients were female. Average BMI was 19.5 ± 5.2 kg/m2, and average BMI Z-score was −0.3 ± 1.9. The most common diagnosis was cerebral palsy (n = 58, 39.5%), followed by other causes (n = 37, 25.1%), muscular dystrophy (n = 16, 10.9%), spinal muscular atrophy (n = 13, 8.8%%), myelodysplasia (n = 11, 7.5%), Rett syndrome (n = 8, 5.4%), and post-traumatic (n = 4, 2.7%). Demographics of the patients in this study are shown in Table 1. Sixty-five (44.2%) patients had any complication in this study. Notably, 17 (11.6%) patients had a deep SSI, 16 (10.9%) had a superficial SSI, 10 (6.8%) developed pneumonia within 30 days, 22 (15.0%) required readmission within 30 days, and 24 (16.3%) required return to the operating room within 90 days. Four patients (2.7%) expired within 1 year postoperatively, with 3 of these occurring within 30 days after surgery. Rates of complications assessed in this study are summarized in Table 2. The distribution of patients across BMI Z-score bands is summarized in Supplementa Table S1.

**Table 1.** Demographics (n = 147)

Age (years)	13.2 ± 3.5
Female sex	83 (56.5%)
BMI (kg/m^2^)	19.5 ± 5.2
BMI Z-score	−0.3 ± 1.9
Diagnosis	
Cerebral palsy	58 (39.5%)
Muscular dystrophy	16 (10.9%)
Spinal muscular atrophy	13 (8.8%)
Myelodysplasia	11 (7.5%)
Rett syndrome	8 (5.4%)
Post-traumatic	4 (2.7%)
Other^a^	37 (25.1%)

BMI, body mass index.

a Includes etiologies such as syrinx, Friedrich ataxia, arthrogryposis, neurofibromatosis, Charcot-Marie-Tooth disease, and other genetic or syndromic conditions.

**Table 2.** Postoperative Complication Rates

30-day mortality	3 (2.0%)
1-year mortality	4 (2.7%)
Deep SSI	17 (11.6%)
Superficial SSI	16 (10.9%)
Loss of signals	16 (10.9%)
Pneumonia within 30 days	10 (6.8%)
30-day readmission	22 (15.0%)
Return to operating room within 90 Days	24 (16.3%)
Return to ER within 90 days	33 (22.4%)
Instrumentation failure within 1 Year	6 (4.1%)

BMI, body mass index; SSI, surgical site infection; ER, emergency room.

### Logistic Regression

**Figure 1.**
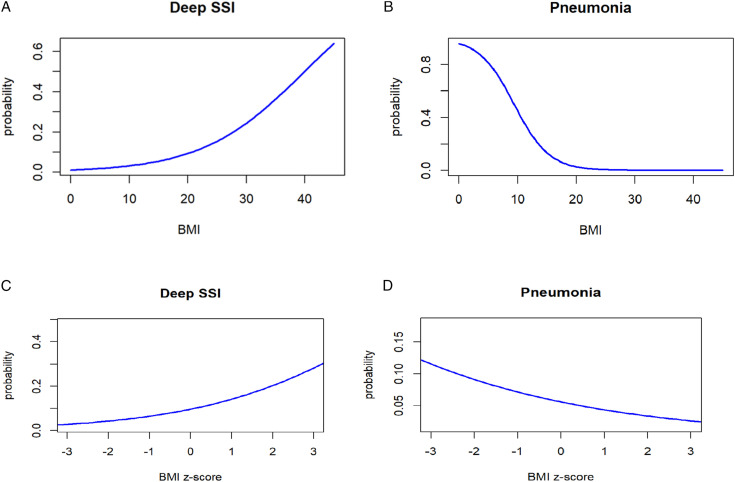
Probability curves from logistic regressions: (A) probability of deep SSI vs BMI, (b) probability of pneumonia vs BMI, (c) probability of deep SSI vs BMI Z-score, (d) probability of pneumonia vs BMI Z-score

A higher BMI Z-score was associated with an increased odds of deep SSI (odds ratio [OR] = 1.50, 95% CI = 1.02-2.20, *P* = 0.039), readmission within 30 days (OR = 1.45, 95% CI = 1.01-2.02, *P* = 0.041), return to the operating room within 90 days (OR = 1.52, 95% CI = 1.07-2.16, *P* = 0.026), as well as a decreased odds of pneumonia (OR = 0.77, 95% CI = 0.59-0.98, *P* = 0.041). Similar findings were also seen for BMI. Odds ratios for all complications between BMI and BMI Z-score can be found in Table 3. Lastly, there was no association between the BMI group and any of the complications assessed (Table 4). Probability curves for deep SSI and pneumonia are shown in Figure 1.

**Table 3.** Logistic Regression of Complications for BMI and BMI Z-Score

	BMI		BMI Z-score	
	OR (95% CI)	*P*-value	OR (95% CI)	*P*-value
30-day mortality	0.88 (0.55-1.16)	0.516	1.09 (0.61-3.07)	0.842
1-year mortality	0.86 (0.64-1.15)	0.312	0.98 (0.65-1.84)	0.945
Deep SSI	1.12 (1.03-1.22)	**0.008**	1.50 (1.02-2.20)	**0.039**
Superficial SSI	1.06 (0.97-1.15)	0.175	1.33 (0.95-1.96)	0.130
Loss of signals	0.95 (0.84-1.07)	0.543	0.98 (0.76-1.31)	0.875
Pneumonia within 30 days	0.72 (0.55-0.92)	**0.008**	0.77 (0.59-0.98)	**0.041**
Readmission within 30 days	1.08 (1.01-1.17)	**0.040**	1.45 (1.01-2.02)	**0.045**
Return to operating room within 90 days	1.11 (1.02-1.20)	**0.011**	1.52 (1.07-2.16)	**0.026**
Return to ER within 90 days	1.04 (0.97-1.11)	0.250	1.19 (0.93-1.51)	0.170
Instrumentation failure within 1 Year	1.12 (0.999-1.25)	0.051	1.71 (0.88-3.33)	0.116

BMI, body mass index; OR, odds ratio; CI, confidence interval; SSI, surgical site infection; ER, emergency room.

*P*-values in bold indicate statistical significance.

**Table 4.** Logistic Regressions of Complications Stratified by BMI Categories^a^

	OR (95% CI)	*P*-value
Deep SSI		
*Underweight*	0.278 (0.015-1.478)	0.226
*Normal*	1.14 (0.389-3.572)	0.814
*Overweight*	0.518 (0.028-2.858)	0.538
*Obese*	3.065 (0.77-10.406)	0.084
Superficial SSI		
*Underweight*	0.257 (0.013-1.356)	0.198
*Normal*	0.96 (0.337-2.834)	0.939
*Overweight*	1.929 (0.407-6.929)	0.348
*Obese*	1.785 (0.378-6.356)	0.406
Pneumonia		
*Underweight*	1.92 (0.393-7.445)	0.368
*Normal*	1.818 (0.483-8.708)	0.401
*Overweight*	1.02 (0.63-1.72)	0.992
*Obese*	1.03 (0.75-1.90)	0.992
30-day readmission		
*Underweight*	0.381 (0.058-1.422)	0.212
*Normal*	1.739 (0.683-4.826)	0.260
*Overweight*	1.10 (0.60-1.98)	0.991
*Obese*	2.534 (0.739-7.707)	0.113
Return to operating room within 90 days		
*Underweight*	0.183 (0.01-0.944)	0.105
*Normal*	1.259 (0.495-3.379)	0.634
*Overweight*	1.333 (0.287-4.604)	0.675
*Obese*	1.882 (0.49-5.998)	0.311
Return to ER within 90 days		
*Underweight*	0.517 (0.143-1.476)	0.256
*Normal*	1.677 (0.757-3.892)	0.212
*Overweight*	0.426 (0.065-1.622)	0.274
*Obese*	1.387 (0.417-4.032)	0.564

BMI, body mass index; OR, odds ratio; CI, confidence interval; SSI, surgical site infection; ER, emergency room.

a BMI categories were defined using CDC BMI-for-age percentiles: underweight (<5th percentile), normal weight (5th to <85th percentile), overweight (85th to <95th percentile), and obese (≥95th percentile).

*P*-values in bold indicate statistical significance.

### Variables Associated with Pneumonia within 30 days Postoperatively

Compared to those who did not develop pneumonia, the ten patients with pneumonia were younger (10.5 vs 13.3 years, *P* = 0.014) and had lower BMIs (15.6 vs 19.8 kg/m2, *P* = 0.013) and BMI Z-scores (−1.6 vs −0.23, *P* = 0.028). Patients who developed pneumonia had high rates of gastrostomy tubes (90.0% vs 42.3%, *P* = 0.003), tracheostomies (40.0% vs 6.6%, *P* < 0.001), chronic pulmonary disease (100.0% vs 62.0%, *P* = 0.015), and history of recurrent pneumonia (50.0% vs 19.7%, *P* = 0.025). Data comparing those with and without pneumonia postoperatively is summarized in Table 5.

**Table 5.** Variables Associated With Pneumonia Within 30 days Postoperatively

	Control (N = 137)	Pneumonia (N = 10)	*P*-value
Age (years)	13.3 ± 3.4	10.5 ± 3.6	**0.014**
BMI (kg/m^2^)	19.8 ± 5.3	15.6 ± 2.6	**0.013**
BMI Z-score	−0.23 ± 1.7	−1.6 ± 3.3	**0.028**
Gastrostomy tube	58 (42.3%)	9 (90.0%)	**0.003**
Tracheostomy	9 (6.6%)	4 (40.0%)	**<0.001**
Chronic pulmonary disease	85 (62.0%)	10 (100.0%)	**0.015**
History of recurrent pneumonia	27 (19.7%)	5 (50.0%)	**0.025**

BMI, body mass index.

*P*-values in bold indicate statistical significance.

## Discussion

The role of BMI in the perioperative optimization of patients with neuromuscular scoliosis undergoing spinal instrumentation has become a common factor in assessing patient outcomes during the surgical decision-making process; however, its actual efficacy remains a subject of ongoing debate. The objective of the present study is to investigate the association between BMI Z-scores and postoperative complications in patients with neuromuscular scoliosis undergoing spinal instrumentation. Our results show that a higher BMI Z-score was associated with increased odds of deep SSI, readmission within 30 days, and return to the operating room within 90 days but a decreased odds of developing pneumonia within 30 days. These findings were corroborated when compared to standard BMI, though no significant findings were observed when patients were categorized into BMI groups of underweight, normal-weight, overweight, and obese.

Our results are consistent with previous studies that examined the relationship between BMI and SSI after neuromuscular scoliosis surgery. Malik et al used the 2012-2016 American College of Surgeons (ACS) National Surgical Quality Improvement Program (NSQIP) database and identified 1291 patients that underwent posterior spinal fusion for neuromuscular scoliosis. This study demonstrated that obese patients had a significantly higher risk of SSI, wound dehiscence, urinary tract infections, and 30-day readmissions compared to normal-weight patients.^9^ Similarly, Basques et al observed that a BMI-for-age ≥95th percentile was associated with a higher risk for infectious complications following PSIF in patients with neuromuscular scoliosis.^5^ Ramo et al analyzed 428 neuromuscular scoliosis patients from a single institution undergoing posterior spinal instrumentation and fusion (PSIF) with a mean 4.9 years follow-up and found that a BMI >25 kg/m2 was associated with a greater risk of SSI.^10^ Lastly, in a matched case-control study by Croft et al, it was shown that the diagnosis of neuromuscular scoliosis and weight-for-age >95th percentile were significant risk factors for developing SSI.^8^

Wound complications, especially SSIs, have been an area of focus in scoliosis correction surgery patients with neuromuscular diseases due to prolonged hospital stays, readmission, need for additional procedures as well as increased financial burden.^15,16^ Different attempts have been made to reduce the risk of wound complications in patients with neuromuscular scoliosis. Plastic-layered closure is a relatively recent practice for these patients at high risk of wound complications. Two studies have shown that plastic-layered closure has been effective in reducing the risk of SSI, although the evidence is still limited due to wide variations in surgical techniques of closure and unclear indications for which patients benefit from this type of closure.^17,18^ This highlights the importance of optimizing BMI and nutrition to decrease the risk of wound complications and SSI. It is important to note that 13 of the 24 patients (54.2%) that required return to operating room within 90 days were for the treatment of deep SSI.

BMI is an important modifiable factor that can be used to optimize a patient’s preoperative surgical risk; however, BMI, even when adjusted for age and height, may be an imperfect measurement of one’s ideal weight, especially for patients with complex medical comorbidities such as those with neuromuscular scoliosis. For example, patients with cerebral palsy (CP) often have significant scoliosis. BMI charts specifically tailored to this population, based on the widely used Gross Motor Functional Classification System (GMFCS) system, have been developed.^19^ Baranek et al showed that the GMFCS-specific BMI may be more predictive of the risk of SSI compared to standard CDC BMI for the subset of patients with CP.^20^ In the present study, we used age- and sex-adjusted BMI Z-scores, and while there were significant associations between the BMI Z-score and BMI with multiple complications, no significant associations were found when comparing outcomes with standard CDC-defined BMI groups. This highlights a possible limitation in the predictive utility of the conventional CDC-defined pediatric BMI scales for those with early-onset scoliosis, as many have other comorbid conditions.

In the present study, our results suggest that patients with a lower BMI Z-score may be at higher risk of developing pneumonia than patients with a higher BMI Z-score. This finding is notable since pulmonary complications are the most common complication after neuromuscular scoliosis surgery, and respiratory failure is the major cause of mortality in patients with neuromuscular disorders.^21,22^ This predisposition is in part due to patients with neuromuscular disorders having poor control and function of respiratory muscles, which worsens with progressive scoliosis.^23^ This problem is further exacerbated with these patients often being underweight and malnourished, and several studies have shown associations with malnutrition and low BMIs with postoperative complications, including pulmonary complications in patients undergoing spine surgery.^24,25,26,27,28,29^ Previous studies have also shown that nutritional status and pulmonary function worsen after scoliosis surgery, which may explain why these patients are at higher risk of complications such as pneumonia postoperatively.^30,31^ Of the ten patients who developed pneumonia postoperatively, these patients were 3 years younger on average and had certain risk factors for developing pneumonia based on our data. Five patients had cerebral palsy, 3 had muscular dystrophy (none were Duchenne muscular dystrophy), 1 had Rett syndrome, and 1 had an unidentified genetic disorder. Nine of these patients had a gastrostomy tube, all ten had chronic pulmonary disease, 5 had a history of recurrent pneumonia, and 4 had tracheostomies. Though we did not include preoperative pre-albumin in this study due to most patients not having this lab value, 8 of the patients who developed pneumonia had this variable measured, with an average value of 16 (range, 7.4 -28.5). The only patient with a normal prealbumin of 28.5 was the patient without a gastrostomy tube. Three of the patients who developed pneumonia died within 30 days postoperatively due to respiratory failure secondary to pneumonia. These data highlight the morbidity of pulmonary complications within this patient population, and those with prior pulmonary disease and malnutrition being at the highest risk. These findings are consistent with results in the literature regarding the association of malnutrition and low BMI with increased complications, including pulmonary complications. Jevesar et al studied forty-four patients with cerebral palsy who underwent scoliosis surgery, and those who were malnourished had higher rates of infection and longer periods of intubation and hospitalization.2^25^ Alsoof, et al found that patients with a BMI of less than 20 had an increased odds of pulmonary complications following lumbar fusion.^24^ Additionally, the same researchers reported that malnutrition, regardless of BMI, was associated with an increased odds of pulmonary complications after posterior lumbar spine fusion.^26^

Investigators have looked to more objective surrogate measurements of nutritional status aside from BMI, such as albumin, prealbumin, and transferrin. A recent study by Furdock and Luhman found no association between preoperative lab values of transferrin, prealbumin, albumin, white blood cell count, total lymphocyte count, and total protein with the development of SSI in neuromuscular scoliosis patients undergoing posterior spinal fusion.^32^ These same authors also did not find an association between preoperative nutritional lab values and postoperative respiratory complications; however, they did identify the presence of gastrostomy tube and history of pneumonia as risk factors.^33^ Similarly, Nishnianidze et al also identified gastrostomy tube dependence being associated with worse postoperative complication scores.^34^ Although further research is needed to identify the best methods for determining a patient’s nutritional status and how to optimize them prior to surgery, a study by Meltzer-Bruhn et al showed that a nutrition consultation did not increase preoperative weight in patients undergoing neuromuscular scoliosis surgery, but gastrostomy tubes were helpful.^27^

Although the present study provides new information about the association between BMI Z-scores and postoperative complications in neuromuscular scoliosis undergoing spinal instrumentation, it has several limitations. First, our study is subject to the limitations inherent to its retrospective design, such as selection bias and residual confounding. Second, this was a single-center study with a relatively small sample size, which may limit generalizability and reduce our ability to differentiate between certain subtypes and populations. In addition, there were low event counts for several complications, including pneumonia, which prevented us from performing multiple logistic regression and thus, limited our ability to control for confounders, including baseline pulmonary status in the pneumonia subgroup. Third, while our study does support the potential use of BMI Z-scores, it is important to note that BMI, which is intrinsic to the BMI Z-score, has been increasingly challenged in the literature as an imperfect metric for assessing body composition and health risks, especially in populations with atypical growth patterns and comorbidities.^35^ Lastly, it is possible that differences in perioperative care and center-specific practice patterns could play some role in our results, including potential surgeon-level or pathway-level effects that were not directly assessed; surgeon case volume and surgeon-level volume effects could not be reliably evaluated in this retrospective cohort. There is literature that states BMI-tailored preoperative protocols can affect outcomes^36,37^; however, this was not assessed in the present study.

## Conclusion

The present study is the first to evaluate the association between BMI Z-scores and postoperative complications in pediatric patients with neuromuscular scoliosis undergoing spinal instrumentation. Our study showed that higher BMI Z-scores were associated with an increased odds of deep SSI, readmission within 30 days, and return to the operating room within 90 days, while lower BMI Z-scores were associated with pneumonia; however, this subgroup had higher rates of baseline pulmonary comorbidity, and thus this finding should be interpreted with caution. BMI and BMI Z-scores had similar performance in this study. There were no differences in complication rates when patients were stratified into CDC-defined BMI-for-age percentiles groups. Our findings suggest that lower BMI Z-scores may help identify a clinically higher-risk subgroup for postoperative pneumonia, potentially reflecting reduced physiologic reserve in medically complex patients, and further studies assessing preoperative nutritional and respiratory optimization would be clinically useful. Despite these findings, additional studies with larger sample sizes, greater statistical power, and additional variables are needed to better assess the performance of BMI Z-scores and identify risk factors for complications in this patient population.

## Supplemental Material


Supplemental material - Association Between BMI Z-Score and Postoperative Complications in Pediatric Neuromuscular Scoliosis Surgery
Supplemental material for Association Between BMI Z-Score and Postoperative Complications in Pediatric Neuromuscular Scoliosis Surgery by Alexander K. Mihas, MD, Dion Birhiray, BS, Chun Wai Hung, MS, MD, Lorenzo Deveza, MD, PhD, Dallas Vanorny, MD, PhD, Frank Gerow, MD, Darrell Hanson, MD, Benny Dahl MD, PhD in Global Spine Journal
