# Supplementary material for: Association Between BMI Z-Score and Postoperative Complications in Pediatric Neuromuscular Scoliosis Surgery
Source: Global Spine J. 2026 Mar 8:21925682261430676. Online ahead of print. doi: 10.1177/21925682261430676 (PMC12971508; doi:10.1177/21925682261430676)
Supplement: Supplemental material - Association Between BMI Z-Score and Postoperative Complications in Pediatric Neuromuscular Scoliosis Surgery [file sj-pdf-1-gsj-10.1177_21925682261430676.pdf]

Supplementary Table S1. Distribution of BMI Z-score bands

BMI Z-score band	n (%)
< −3	9 (6.1%)
−3 to < −2	11 (7.5%)
−2 to < −1	20 (13.6%)
−1 to < 0	35 (23.8%)
0 to < +1	36 (24.5%)
+1 to < +2	27 (18.4%)
≥ +2	9 (6.1%)
Total	147 (100.0%)

BMI, body mass index.
